# Psychosocial job conditions, fear avoidance beliefs and expected return to work following acute coronary syndrome: a cross-sectional study of fear-avoidance as a potential mediator

**DOI:** 10.1186/s12889-015-2599-z

**Published:** 2015-12-21

**Authors:** Mia Söderberg, Annika Rosengren, Sara Gustavsson, Linus Schiöler, Annika Härenstam, Kjell Torén

**Affiliations:** Department of Occupational and Environmental Medicine, Institution of Medicine, Sahlgrenska Academy & University of Gothenburg, Box 414, 405 30 Gothenburg, Sweden; Institute of Medicine, Sahlgrenska Academy, University of Gothenburg, SU/Östra, Gothenburg, Sweden; Department of Sociology and Work Science, University of Gothenburg, Gothenburg, Sweden; Department of Psychology, Stockholm University, Stockholm, Sweden

**Keywords:** Job demand-control, Effort-reward imbalance

## Abstract

**Background:**

Despite improvements in treatment, acute coronary syndrome remains a substantial cause for prolonged sick absences and premature retirement. Knowledge regarding what benefits return to work is limited, especially the effect of psychological processes and psychosocial work factors. The purposes of this cross-sectional study were two-fold: to examine associations between adverse psychosocial job conditions and fear-avoidance beliefs towards work, and to determine whether such beliefs mediated the relationship between work conditions and expected return to work in acute coronary syndrome survivors.

**Methods:**

Study inclusion criteria: acute myocardial infarction or unstable angina diagnosis, below 65 years of age, being a resident in the West county of Sweden and currently working. In all, 509 individuals (21.8 % women) accepted study participation and for whom all data of study interest were available for analysis. Psychosocial work variables; job demand-control and effort-reward imbalance, were assessed with standard questionnaire batteries. Linear regression models were used to investigate relationships between psychosocial factors and fear-avoidance, and to evaluate mediator effects for fear-avoidance. Both total sample and gender stratified analyses were calculated.

**Results:**

Fear-avoidance beliefs about work were associated to psychosocial job environments characterized by high strain (β 1.4; CI 1.2–1.6), active and passive work and high effort-reward imbalance (β 0.6; CI 0.5–0.7). Further, such beliefs also mediated the relationship between adverse work conditions and expected time for return to work. However, these results were only observed in total sample analyses or among or male participants. For women only high strain was linked to fear-avoidance, and these relationships became non-significant when entering chosen confounders.

**Conclusions:**

This cross-sectional study showed that acute coronary syndrome survivors, who laboured under adverse psychosocial work conditions, held fear-avoidance beliefs towards their workplace. Furthermore, these beliefs mediated the relationships between - high strained or high effort-reward imbalanced work - and expected return to work. However, mentioned results were primarily found among men, which could results from few female study participants or gender differences in return to work mechanisms. Still, an earlier return to work might be promoted by interventions focusing on improved psychosocial work conditions and cognitive behavioural therapy targeting fear-avoidance beliefs.

## Background

Advances in the treatment of acute coronary syndromes (ACS), e.g. pharmacological treatment and revascularisation procedures, have increased survival and augmented the numbers of ACS survivors in the work force [1]. These advances have, however, not been reflected in improved rehabilitation outcomes, as ACS remains a widespread cause for extended work absences [1, 2] and premature retirement [3]. Prolonged sick-leave carries several disadvantages; social isolation and weakened financial position, as well as economical societal consequences due to productivity loss [4]. Previous studies, investigating dimensions that promote return to work (RTW) among cardiovascular heart disease patients (CHD), have focused on factors such as disease severity, socioeconomics, age, and physical work demands [3, 5, 6]. Few studies have investigated associations to psychosocial work conditions or how perceptions of the work environment are linked to RTW. The lack is noteworthy, since one review study [5] found that psychosocial factors, in particular job stress and the perception of not being able to cope with job stress, were stronger predictors for work resumption than disease severity.

The two most evaluated models for measuring stressing psychosocial job conditions, are the job demand-control (JDC) [7] and effort-reward imbalance (ERI) models [8]. In the former construct, the “demand” variable captures psychological work load, while “control” measures the employee’s influence over the content and volume of work tasks. Commonly these two variables are dichotomized into high/low and combined to form four different categories of work environments [9], *high strain* (high demand-low control), *active* (high demand-high control), *passive* (low demand-low control) and *low-strain* (low demand-high control)*.* The ERI model measures reciprocity between effort and reward. Conceptually, “effort” is similar to job demand in measuring work intensity, while “reward” captures esteem from colleagues and management, salary, career opportunities and job security. “Imbalance” reflects a state where demands are disproportionate to associated rewards. Throughout the literature high strain and effort-reward imbalance, has frequently been linked to CHD [10–12]. Additionally, some studies have also observed associations to recurrent myocardial infarction [13, 14]. Despite this evidence, only one study found evaluated links between these psychosocial variables and RTW after ACS [15]. Results showed that high strain was an independent predictor for prolonged RTW, even after adjusting for sex, socioeconomics, smoking and depression. Furthermore, regardless of proven relationships between work variables and ill-health, few studies investigate how workers themselves interpret adverse job conditions. This lack of research is surprising, as it seems improbable that workers are passive recipients, who do not reflect upon or try to protect their health, by altering or avoiding hazardous job conditions.

A prominent predictor for prolonged sick absence in musculoskeletal conditions, is fear-avoidance beliefs towards work [16, 17]. Fear-avoidance is characterized by negative attribution of work as hazardous to health, leading to fear of further exposure and the interpretation that avoiding work equals reduction of harm [18]. Fear-avoidance beliefs have not been previously studied among CHD patients. However, a review paper investigating “casual attribution” i.e. what CHD patients believed caused their disease [19], found that chronic stress was the most common attributed cause, even higher than lifestyle habits and obesity. In another study among myocardial infarction patients, life stress was also the most frequent cited cause [20]. Although work is a dominant source for stress in most modern societies [21], only a handful studies investigated if patients attributed their disease to stressing psychosocial work conditions. Some papers reported attributions to “overwork” [19], but the definition of this concept was broad and could be defined as both time at work or physical effort in or outside the workplace. In sum, detrimental work factors might not be independently related to prolonged sick-absence, but rather how these conditions are perceived, and associations with fear and the beliefs that resuming work will exacerbate or lead to recurrent ACS. This study therefore aims to investigate (1) associations between adverse psychosocial job dimensions and fear-avoidance beliefs and (2) whether fear-avoidance mediated the relationship between psychosocial work environment and expected RTW in ACS survivors.

## Method

### Study population

**Fig. 1:**
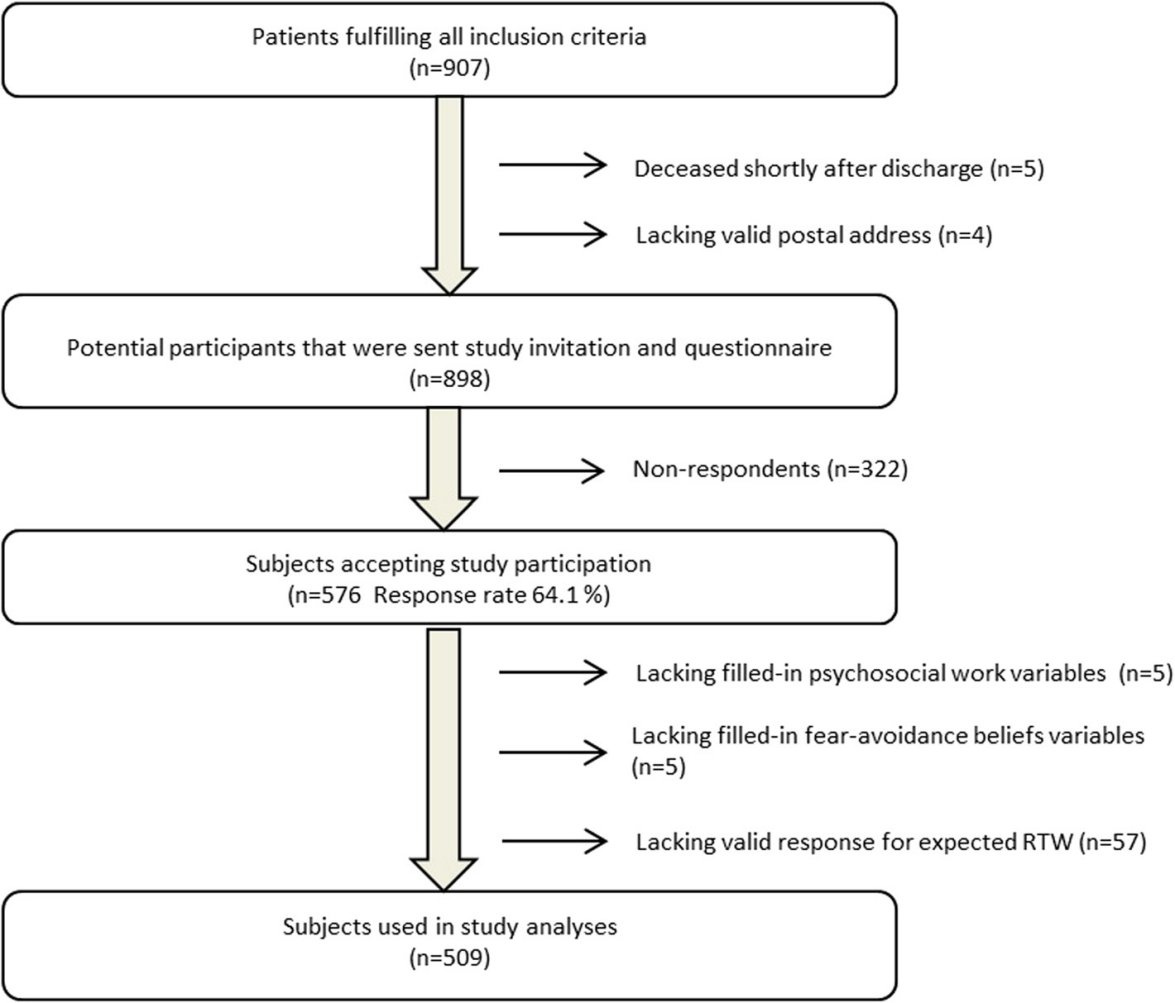
Flowchart illustrating selection process for studied sampleᅟ

Study participants were recruited from the VGR-heart study (VGR = Västra Götalands Regionen i.e. West county of Sweden). The VGR-heart project is a population based cohort study which aims to identify occupational predictors for RTW after ACS. Data collection was carried out December 2010 to December 2013. Inclusion criteria were: acute myocardial infarction or unstable angina diagnosis, an upper age of 65 years, being a resident of the VGR and currently employed in paid work or self-employed. An upper age of 65 was chosen since it’s the Swedish general retirement age, consequently older individuals are unlikely to work or prone to retire, rather than continue working if suffering from ACS onset. Screening for participants took place at four hospitals: Sahlgrenska University hospital, Östra hospital, Skaraborg hospital and North Älvsborg county hospital. Due to administrative circumstances the North Älvsborg county hospital only participated in subject recruitment: March 2011–March 2013. In total, 907 patients fulfilled the inclusion criteria. Five individuals died shortly after discharge and four lacked a valid postal address, and thus 898 individuals were sent one questionnaire and a consent form, allowing hospital record and register data collection. A total of 576 individuals accepted study participation, representing a response rate of 64.1 %. Some participants completely lacked filled-in items for psychosocial dimensions (*n* = 5), fear-avoidance (*n* = 5) or expected time for RTW (*n* = 57) and were omitted; the final sample consisted of 509 subjects (illustrated in Fig. 1). This cross-sectional study has been performed in compliance with the Helsinki Declaration, followed the STROBE statement guidelines and was approved by the Regional Ethical Review board of Gothenburg.

### Questionnaire

The questionnaire recorded basic demographics, occupational history, shift work patterns, smoking, psychosocial work conditions, work ability, personality variables, fear-avoidance beliefs towards work and general mental health.

*Job demand-control* was measured using the Swedish version of Karasek & Theorell’s Job Content Questionnaire, labelled The Swedish Demand—Control—Support Questionnaire (DCSQ) [22]. For the purposes of this analysis, demand and control variables were positively inverted so that high scores equated with high demands or high control. Both measures were tallied separately; summary scores ranged from 5 to 20 (job demand) and 9–24 (job control). Median scores for demand and control were 13 and 19, respectively. Each variable was dichotomized into high or low by the median values of the distributions. The dichotomized variables were combined into categories: *high strain* (high demand-low control), *active* (high demand-high control), *passive* (low demand-low control) and *low strain* (low demand-high control)*.*

The reward variable in *effort-reward imbalance,* was assessed using a standard instrument, the Effort-Reward Imbalance at Work Questionnaire [23]. Effort was replaced with the five items used to measure job demands, as these two variables have proven to capture similar dimensions [24]. All reward items were positively inverted and then tallied. Sum reward scores ranged 14–50 (median = 45.0). According to a standard algorithm, a ratio value was created (Σ_effort_/(Σ_reward*0.4545)_). The observed ERI-ratio values ranged from 0.2 to 2.1. Given the narrow distribution range, we wanted to specify levels for the ratio, or else the levels would be set to 1.0 by default. In some studies, categorization is divided by the quartiles, but since the ERI-ratio distribution in our sample was skewed towards lower scores, we decided to specify levels per 0.25 of the distribution.

*Fear-avoidance* was captured by five items from the Fear-avoidance Beliefs Questionnaire [25] and one item from the Obstacles for Return to Work Questionnaire in chronic pain [26]. The likert-type response option scale for all items ranged from “Completely disagree” to “Completely agree,” scored 1–6. The original instruments focused on pain in relation to physical activities; hence items were rephrased for this study to better suit heart disease conditions. Original and remodelled items can be found in Appendix A. In order to evaluate the new measure, we assessed internal consistency using Cronbach’s alpha, yielding a score of 0.89. We further evaluated coherence, using factor analysis. All six items loaded strongly on the first factor, with a sharp fall-off in eigenvalue after that, consistent with a battery reflecting one domain. Fear-avoidance was then converted into an index based on each participant’s mean score. The index ranged 1–6 (mean value = 2.3, SD = 1.2). The mean, instead of the median was used, since we wanted to allow extreme values to have an impact on the index.

To measure the outcome variable, *expected time for return to work*, one single item was used: “Based on everything you know and feel now, when do you think you will be able to return to work? Estimate the amount of weeks”. This amount was then added together with the response time, i.e. time elapsed between hospital discharges and the date for filled in questionnaire. Some subjects (*n* = 13) had already returned to work when filling-in the questionnaire, but had provided information on time on sick leave. Although this measures actual time for RTW, the information was incorporated with expected time for RTW.

Occupational status was measured with one item, classified according to ISCO-88 [27] and categorized into three main occupational categories: white-collar (e.g. executives, managers, professionals), pink-collar (female dominated jobs e.g. office and health service workers) and blue-collar workers (e.g. plant and machine operators, jobs without formal training). *Self-efficacy* was captured with two items: “Once I’ve decided to return to my job, it won’t be difficult for me to accomplish this” and “Despite what has happened, I know that I’ll manage to carry out my work when I feel well enough”, scale ranging (1–4) from “Completely disagree to “Completely agree”. For measurements of *mental health* the 12-item General health questionnaire (GHQ-12) [28] was used.

### Statistical analysis

Statistical calculations were carried out with SAS version 9.2 for Windows (SAS Institute; Cary; NC). All missing items for demand, control, reward and fear-avoidance scores were imputed. Imputed values were based on each participant’s mean scores of the remaining items in each variable.

For our linear regression analyses we considered the following confounders: age, occupational status, pre-morbid work ability, social support at the work, self-efficacy, attitudes towards sick leave and general mental health, factors which all previously have been associated to RTW [29–31]. All covariates were examined for co-linearity, using Spearman correlations analyses due to the categorical properties of some variables. Correlation coefficient values >0.4 was considered as co-linearity. Social support was correlated to both demand and ERI-ratio (r = −0.50 and −0.43 respectively), and was excluded from further analyses. Pre-morbid work ability displayed co-linearity with general mental health. Based on previous studies illustrating the importance of mental health for RTW among CHD survivors [5, 15], mental health was chosen as a confounder in our analyses.

To further determine which confounders to include in our model, we utilized stepwise purposeful selection as proposed by Hosmer & Lemeshow [32], with separate calculations for fear-avoidance perceptions and RTW as outcome variables. Inclusion cut-off was F-test *p*-value <0.25. The selection process began with univariate analyses of each variable, where all variables meeting the cut-off constituted the full model. These variables and main psychosocial measures were then entered together in linear regression analyses. Variables with p-value >0.25 were excluded. The excluded variables were then added back one at a time and reinserted into the model if meeting inclusion criteria. The remaining variables constituted the reduced model. Differences in main effects between the reduced model and full model were less than 15 %, hence the reduced model was kept. After concluding the selection process the following confounders remained; *occupational status*, *self-efficacy, and general mental health.*

For the main analyses, linear regression models were used to investigate associations between psychosocial variables and fear-avoidance beliefs, and mediation effects for fear-avoidance. The categories, high strain, active, passive and low strain, were converted into dummy variables, using low strain as reference. When analysing ERI, specified levels per 0.25 of the ERI-ratio distribution was set. The fear-avoidance index and time for RTW were entered as continuous variables. In order to investigate impact of confounders, two models were calculated. Model 1 was unadjusted and Model 2 was adjusted for occupational status, self-efficacy and general mental health. JDC and ERI as were analysed separately. All models were stratified by gender.

**Fig. 2:**
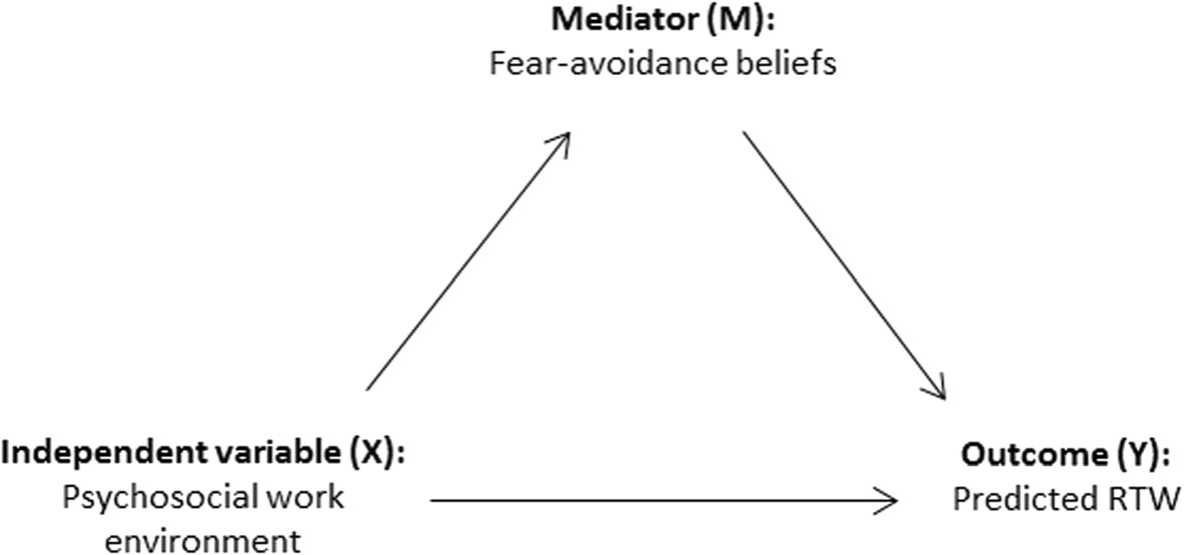
Stepwise procedures for mediation testingᅟ

To evaluate mediator effects for fear-avoidance, a four step procedure [33] was used (Fig. 2). Three linear regression analyses were assigned to explore relationships between direct effects; psychosocial variables to fear-avoidance (X→M): psychosocial factors to RTW (X→Y), and fear-avoidance to RTW (M→Y). Should all associations in step 1–3 prove significant, a fourth linear regression analysis is carried out where psychosocial variables and mediator are entered in the same model (X+M→Y). If the effect for the mediator (M) remains significant, there is support for partial mediation, and if associations for the psychosocial variables (X) simultaneously become non-significant, the findings support full mediation.

## Results

Sample characteristics; basic demographics, psychosocial variables, fear-avoidance beliefs index and time for return to work, are found in Table 1. To clarify which subgroups that fulfilled mediation testing requirements, and thus would be included in the final analyses (X+M→Y); subgroups with non-significant associations between direct effects were omitted in the following step.

**Table 1:** Age, occupational status, smoking, psychosocial variables, fear-avoidance and time for return to work

	All		Men		Women	
	N	%	N	%	N	%
Number of participants	509	100	398	78.2	111	21.8
White-collar, N (%)	175	42.2	140	43.9	35	36.5
Pink-collar, N (%)	84	20.2	38	11.9	46	47.9
Blue-collar, N (%)	156	37.6	141	44.2	15	15.6
Non-smoker	174	34.4	148	37.3	26	23.9
Ex-smoker, N (%)	166	32.8	136	34.3	30	27.5
Current smoker, N (%)	166	32.8	113	28.5	53	48.6
High strain, N (%)	163	32.0	108	27.1	55	49.6
Active, N (%)	59	11.6	51	12.8	8	7.2
Passive, N (%)	141	27.7	110	27.6	31	27.9
Low strain, N (%)	146	28.7	129	32.4	17	15.3
	Mean	SD	Mean	SD	Mean	SD
Age	55.9	5.9	56.0	5.8	55.4	6.3
ERI-ratio	0.6	0.2	0.6	0.2	0.7	0.3
Fear-avoidance beliefs	2.3	1.1	2.3	1.1	2.4	1.2
Time for RTW (in weeks)	7.1	5.1	6.9	5.1	7.8	5.2

Linear regression analyses for psychosocial work conditions and fear-avoidance beliefs (X→M) (Table 2) showed that in unadjusted total sample analyses, subjects with high strain (β 1.4; CI 1.2–1.6), active (β 0.6; CI 0.3–0.9) or passive jobs (β 0.4; CI 0.2–0.6), reported higher fear-avoidance than low strain workers. The associations remained in model 2, although estimates were slightly lower. The results among male subjects displayed similar results. In female participants, only high strain work was linked to increased fear-avoidance, and results became non-significant when entering chosen confounders. High ERI was related to increased fear-avoidance scores in all analyses and for both model.

**Table 2:** Linear regression analyses between psychosocial variables and fear-avoidance (X → M)

	All		Men		Women	
	β (95 % CI)	*p*-value	β (95 % CI)	*p*-value	β (95 % CI)	*p*-value
Job demand-Control						
Model 1						
High strain	**1.4 (1.2–1.6)**	**<.0001**	**1.6 (1.3–1.8)**	**<.0001**	1.0 (0.4–1.6)	0.07
Active	**0.6 (0.3–0.9)**	**0.0002**	**0.7 (0.4–1.0)**	**<.0001**	0.3 ((−0.6)–1.3)	0.9
Passive	**0.4 (0.2–0.6)**	**0.02**	**0.5 (0.2–0.7)**	**<.0001**	0.1 ((−0.6)–0.7)	0.3
Low strain (REF)	1.0		1.0		1.0	
Model 2						
High strain	**1.2 (0.9–1.4)**	**<.0001**	**1.3 (1.1–1.6**)	**<.0001**	0.5 ((−0.04)–1.1)	0.07
Active	**0.5 (0.3–0.8)**	**0.0002**	**0.6 (0.3–0.9)**	**<.0001**	0.04 ((−0.8)–0.9)	0.9
Passive	**0.3 (0.03–0.5)**	**0.02**	**0.4 (0.1–0.6)**	**0.002**	−0.3 ((−0.9)–0.3)	0.3
Low strain (REF)	1.0		1.0		1.0	
Effort-reward imbalance						
Model 1	**0.6 (0.5–0.7)**	**<.0001**	**0.7 (0.6–0.8)**	**<.0001**	**0.6 (0.4–0.8)**	**<.0001**
Model 2	**0.5 (0.4–0.6)**	**<.0001**	**0.6 (0.5–0.7)**	**<.0001**	**0.4 (0.2–0.6)**	**0.0003**

95 % CI = 95 % confidence interval

Model 1 Unadjusted; Model 2 Adjusted for Occupational status, self-efficacy and general mental health

Step two in the mediation testing (X→Y) (Table 3), showed that in model 1, highly strained workers reported longer expected time for RTW than participants with low strain, in all subjects analyses (β 2.8; CI 1.7–4.0) and among men (β 3.1; CI 1.8–4.4). High ERI was also related to RTW in unadjusted total sample (β 1.1; CI 0.6–1.5) and male subgroup analyses (β 1.1; CI 0.5–1.6). These effects remained, but with slightly lowered estimates in model 2. There were no significant associations between psychosocial job variables and predicted time for RTW among women.

**Table 3:** Linear regression analyses between psychosocial variables and expected RTW in weeks (X→Y)

	All		Men		Women	
	β (95 % CI)	*p*-value	β (95 % CI)	*p*-value	β (95 % CI)	*p*-value
Job demand-control						
Model 1						
High strain	**2.8 (1.7–4.0)**	**<.0001**	**3.1 (1.8–4.4)**	**<.0001**	1.6 ((−1.2)–4.5)	0.3
Active	0.7 ((−0.8)–2.2)	0.4	0.5 ((−1.1)–2.2)	0.5	1.7 ((−2.7)–6.1)	0.4
Passive	**1.3 (0.1–2.5)**	**0.03**	**1.3 (0.01–2.6)**	**0.05**	0.9 ((−2.2)–4.0)	0.6
Low strain (REF)	1.0		1.0		1.0	
**Model 2**						
High strain	**2.1 (1.0–3.2)**	**0.003**	**2.4 (1.1–3.6)**	0.0002	0.7 ((−2.3)–3.6)	0.7
Active	0.6 ((−0.8)–2.1)	0.4	0.5 ((−1.0)–2.1)	0.8	−0.3 ((−4.6)–4.0)	0.9
Passive	0.9 ((−0.2)–2.0)	0.6	1.0 ((−0.2)–2.3)	0.1	−0.1 ((−3.2)–3.0)	0.9
Low strain (REF)	1.0		1.0		1.0	
Effort-reward imbalance						
Model 1	**1.1 (0.6–1.5)**	**<.0001**	**1.1 (0.5–1.6)**	0.0001	0.8 ((−0.1)–1.8)	0.09
Model 2	**0.7 (0.3–1.2)**	0.002	**0.8 (0.2–1.3)**	0.006	0.3 ((−0.7)–1.3)	0.5

95 % CI = 95 % confidence interval

Model 1 Unadjusted; Model 2 Adjusted for Occupational status, self-efficacy and general mental health

The analyses for relationships between fear-avoidance and RTW (M→Y), not presented in a table, displayed significant associations between fear-avoidance and prolonged RTW, in total sample analyses (β 1.4; CI 95 % 1.0–1.7) and among men (β 1.4 CI 95 % 1.0–1.8).

In the final regression analyses (X+M→Y), evaluating mediator effects for fear-avoidance (Table 4), results supported full mediation effects for fear-avoidance between both high strain and high ERI to RTW, when analysing all sample subjects or males, and for both models.

**Table 4:** Multiple linear regression testing mediator effects for fear-avoidance beliefs (M) between psychosocial variables (X) and expected time for RTW (Y) 95 % CI = 95 % confidence interval

	All		Men	
	β (95 % CI)	*p*-value	β (95 % CI)	*p*-value
Job demand-Control				
Model 1				
High strain	1.2 ((−0.1)–2.4)	0.07	1.2 ((−0.2)–2.7)	0.1
Active	−0.2 ((−1.5)–1.5)	0.9	−0.3 ((−1.9)–1.3)	0.7
Passive	0.8 ((−0.3)–2.0)	0.1	0.7 ((−0.5)–2.0)	0.2
Low strain (REF)	1.0		1.0	
Fear-avoidance	**1.2 (0.7–1.6)**	**<.0001**	**1.2 (0.7–1.7)**	**<.0001**
Model 2				
High strain	1.1 ((−0.1)–2.4)	0.07	1.3 ((−0.1)–2.7)	0.08
Active	0.2 ((−1.3)–1.6)	0.8	0.03 ((−1.5)–1.6)	0.9
Passive	0.7 ((−0.4)–1.8)	0.2	0.7 ((−0.5)–1.9)	0.2
Low strain (REF)	1.0		1.0	
Fear-avoidance	**0.8 (0.4–1.3)**	**0.0003**	**0.8 (0.3–1.3)**	**0.002**
Effort-Reward imbalance				
Model 1	0.3 ((−0.3)–0.8)	0.3	0.2 ((−0.4)–0.8)	0.6
	**1.2 (0.8–1.7)**	**<.0001**	**1.3 (0.8–1.8)**	**<.0001**
Model 2	0.3 ((−0.3)–0.8)	0.3	0.2 ((−0.4)–0.8)	0.5
	**0.9 (0.5–1.4)**	**<.0001**	**0.9 (0.4–1.5)**	**0.0004**

Model 1 Unadjusted; Model 2 Adjusted for Occupational status, self-efficacy and general mental health

## Discussion

The results in our study displayed full mediation effect for fear-avoidance beliefs towards work, in the relationship between adverse psychosocial work conditions - high strain and high effort-reward imbalance - and expected time for return to work among ACS survivors. Moreover, high strain, active and passive work and high ERI, was related to fear-avoidance beliefs. These associations only occurred in total sample analyses or among men, where similarities are likely due to a majority of male participants (78.2 %). In female subjects, fear-avoidance beliefs were related to highly strained and effort-reward imbalanced jobs, but no mediator effects were observed. The results are partly consistent with similar studies among musculoskeletal patients [16, 34], although these studies were not gender stratified, nor did they investigate mediating effects for fear-avoidance in a similar design as the present study.

Several types of psychosocial conditions were related to fear-avoidance beliefs (X → M), in whole sample analyses or among male participants. Again, significant findings were sparse among female participants, as only women reporting high strain seemed to hold fear-avoidance beliefs, and associations became non-significant in the adjusted model. Plausible explanations can be found among gender theory-based studies [35], showing that men to a larger degree attribute harmful events to job stress or unfair treatment, while women are more prone to self-blaming or attribute ill-health to private life stressors. Furthermore, women are more frequently subjected to “double exposure” [36]; a combination of work and private life stress, where e.g. children care [37] or marital issues [38], implied greater stress than occupational factors. Hence, women are both exposed to, and therefore attribute their ill-health to, a wider variety of causes than men, thus deflating relationships between fear-avoidance and work conditions. Women’s’ complex life situation could furthermore explain non-significant results when entering confounders, such as general mental health.

An additional finding was that men with high strain and high ERI expected longer time for work resumption (X → Y); while for women effects were both low and non-significant. Although this particular analysis was not part of the aims of this study, the lack of effects for adverse work conditions among women is worth noting. Results could be due to few female participants (*n* = 124), but as previously discussed, that females are exposed to a larger variety of stress sources [37, 38]. One paper supports that RTW among females is a more complex process, involving simultaneously managing both work and private life [35]. A large Danish study [39], additionally showed that female ACS survivors, had lower rates of RTW than men, and thus further strengthening the argument that gendered differences in RTW needs to be investigated further.

The mediation test (Step 4: X+M → Y) supported full mediation effects for fear-avoidance beliefs between psychosocial factors and RTW. No previous studies have evaluated fear-avoidance beliefs among ACS survivors or mediator effects between job conditions and RTW. A substantial amount of studies in musculoskeletal pain disorder have found that these beliefs are more important for RTW than the actual work conditions [16, 17, 34]. There is also evidence that long-term sick leave, with consequent lack of confrontation and adjustment of such beliefs, can result in near phobic behaviour, with severely prolonged time for work assumption [18]. Based on such evidence, it is common practice to emphasize that the patient remains at work or to promote prompt work resumption in musculoskeletal pain patients. We therefore suggest that further studies should be made examining similar effects for fear-avoidance beliefs and time for returning to work among ACS survivors.

### Limitations

We recognize several potential limitations. A primary limitation was the measurement for time for RTW, since it was based on the participants’ subjective evaluations. However, one review paper in non-chronic non-specific low back pain patients [40] and a longitudinal study studies based on patients with psychiatric disorders [41], showed that expected RTW predicted actual RTW time. Additionally, the VGR-heart project is a longitudinal study, thus exact time for RTW will be captured in future studies with register data from the Swedish national social insurance agency (Försäkringskassan). In the analyses there were 13 participants with actual RTW time scores. Exploratory calculations that excluded these participants displayed very minor differences in results. Since not notably affecting results and not wanting to exclude participants from a fairly small sample, we decided to keep these participants in our analysis.

We further acknowledge that recent ACS onset is likely to affect psychological well-being and results in overestimation of work load, especially depressed individuals, who have been shown to inflate self-reported job demand [42]. We have adjusted our calculations for general mental health (GHQ-12), but it is possible that more confounders capturing mental health dimensions should have been used. The lack of measures for off-work stressors, e.g. child care and household duties, was another limitation, especially considering the strong interplay between these stressors and occupational factors in RTW processes for women.

Yet another source of potential bias was the difference in response time. Previous papers have shown that exposure training and gradual RTW in back-pain patients, lower fear-avoidance attributions over time [43]. Thus those with short response time might therefor be inclined to higher fear-avoidance perceptions, than those already in rehabilitation training. Since response time was part of our outcome measure we could not use that variable as a confounder.

On methodological limitation is that the standard items for effort were not used. However, it has been shown that the demand items from the standard JDC and ERI at work questionnaires are highly related and load strongly on the same factor in factor analyses [24]. Further, while there is some overlap between the two models with respect to the work load intensity, the combination of either job control or rewards, separates the two models and have shown to capture complementary aspects of the work environment [24].

A cross-sectional study is not ideal for attempting to draw causal conclusion. This is because a key element in causal relationships is the time aspect. The exposure must come before the mediator effect, which in turn should come before the outcome. Even if this is a cross-sectional study, attempts have been taken to try to include an approximated time aspect. The job demand-control and effort-reword imbalance were question about the past job situation, i.e. the situation before the event, while fear-avoidance is the participants present state of mind and expected return to work is a prediction of a future event. Some major limitations of this approach, compared to a longitudinal study with three points of measurements, as suggested by Lockhart and colleagues [44], are that the present state of mind might very well create bias in both the recall of the past job situation and the expected return to work. People that feel worse might have a less positive recall of the job situation and a more pessimistic prediction of the expected return to work, which would produce an overestimation of the mediator effect of fear-avoidance.

In order to evaluate external validity, we gathered data on non-respondents from Statistics Sweden based on Swedish personality identity numbers (Table 5). The data obtained showed that those declining participation displayed similar gender proportions and mean age as the subjects in our sample. The most notable deviation is an overrepresentation of white-collar workers among the subjects in our sample, which weakens this study’s external validity, as socioeconomic has been proven to be an important factor for both ACS onset and return to work [45].

**Table 5:** Descriptives of participants and non-respondents

	Participants	Non-respondents
Number of individuals	568	322
Percentage of women	21.3 %	23.0 %
Age, mean	55.7 (6.0)	54.0
White-collar work	192 (41.7)	100 (29.1)*
Pink-collar work	93 (20.2)	73 (21.2)*
Blue-collar work	175 (38.0)	143 (41.6)*

* Statistics Sweden lacked occupational information on some of the non-respondents, hence missing values

## Conclusions

Despite mentioned limitations, this study contributes with knowledge regarding how ACS survivors with adverse psychosocial work conditions might hold fear-avoidance attribution towards the workplace. The findings that the fear-avoidance beliefs mediated relationships between poor job environments and expected return to work, indicates that targeting such attributions are an important factors for effective RTW. Since several covariates were tested, our results highlight the particular importance of adverse work dimensions and related fear-avoidance beliefs in relation to prolonged time for return to work. Although further studies are needed, especially analyses that explore gender differences in attribution and RTW processes, we hope that these findings will provide new the understanding of psychological factors that might decrease time for RTW.

## Appendix A

Fear-avoidance beliefs about work and heart disease used in this study

1. My heart condition has been caused by my work or something that happened at work
2. My work would make my condition worse
3. My work is too heavy for me
4. I should not do my normal work as I did before I fell ill with heart disease
5. My job is detrimental to my health
6. If I had had a another kind of job my heart disease would never have occurred

Corresponding items from the Fear-avoidance Beliefs Questionnaire (1–5) [25] and The reduced items for obstacles for return to work questionnaire (6) [26]

1. My pain was caused by my work or by an accident at work
2. My work makes or would make my pain worse
3. My work is too heavy for me
4. I should not do my normal work with my present pain
5. My work aggravated my pain
6. If I had had another kind of job I would never have gotten any pain
